# Fatal visceral disseminated varicella zoster virus infection during initial remission induction therapy in a patient with lupus nephritis: a case report and review of the literature

**DOI:** 10.1007/s13730-024-00950-7

**Published:** 2024-12-12

**Authors:** Runa Takehara, Itaru Ebihara, Yoshifumi Honda, Norimasa Ooba, Hiromi Kurosawa, Chihiro Sato, Haruo Ohtani, Yutaka Tsutsumi, Masato Nose, Masaki Kobayashi

**Affiliations:** 1 Department of Nephrology, Mito Saiseikai General Hospital, 3-3-10 Futabadai, Mito, Ibaraki 311-4198 Japan; 2 Department of Pathology, Mito Saiseikai General Hospital, Mito, Ibaraki Japan; 3 Pathos Tsutsumi (Tsutsumi Byori Shindanka Clinic), Inazawa, Aichi Japan; 4 Ehime University School of Medicine, Ehime, Japan; 5 Department of Nephrology, Tokyo Medical University Ibaraki Medical Center, Ibaraki, Japan; 6 Ooba Internal Medicine Clinic, Ibaraki, Japan

**Keywords:** Varicella zoster virus, Visceral disseminated varicella zoster virus infection, Lupus nephritis

## Abstract

Visceral disseminated varicella zoster virus (VZV) infection is a severe complication, characterized by a notably high mortality rate. Herein, we present a case of a 36-year-old-man involving visceral disseminated VZV infection that emerged during remission induction therapy involving high-dose prednisolone (PSL), mycophenolate mofetil (MMF), and hydroxychloroquine for lupus nephritis. Two months after starting the immunosuppressive therapy, he experienced a rapid onset of severe upper abdominal pain. The following day, clinical manifestations and laboratory abnormalities rapidly deteriorated. Hyperferritinemia and hypertriglyceridemia, indicative of hemophagocytic lymphohistiocytosis (HLH), emerged, along with escalating liver and renal impairment and newly appeared disseminated intravascular coagulation, and multiple organ failure is suggested. The patient developed widespread blistering predominantly on the trunk and face, patient’s condition failed to ameliorate, ultimately culminating in his demise a few hours later. At autopsy, the cutaneous lesions with blisters revealed positive immunostaining with anti-VZV antibody, and similar findings were detected in multiple organs. HLH was confirmed in lymph nodes. It is crucial to emphasize the awareness of visceral disseminated VZV, particularly in cases patients are undergoing concurrent PSL therapy alongside MMF for SLE. The progression of this fatal condition usually begins with abdominal pain, followed by a skin rash a few days later. The present case is the first to show evidence of HLH occurring as a result of visceral disseminated VZV infection. This disease is extremely rare but extremely serious, therefore, VZV-DNA should be measured in cases where you suspect this disease for early diagnosis and treatment.

## Introduction

Visceral disseminated varicella zoster virus (VZV) infection commonly occurs in immunocompromised individuals. Among this population, the most severe complication is visceral disseminated VZV infection, characterized by a notably high mortality rate. This condition is observed in immunocompromised patients who are undergoing immunosuppressive therapy for hematologic disorders, recipients of kidney transplants, individuals with uncontrolled diabetes, and those with collagen and/or renal disease [1–14]. Therefore, prompt diagnosis and timely treatment are imperative for the survival of these patients.

Visceral disseminated VZV infection has the potential to lead to serious conditions such as hepatitis, pancreatitis, pneumonia, encephalitis, and disseminated intravascular coagulation (DIC), often manifesting with abrupt abdominal pain [4–8]. The increasing variety of immunosuppressive therapies underscores the importance of recognizing visceral disseminated VZV infections and implementing appropriate preventative strategies to avert fatalities in immunocompromised populations.

Herein, we present a case involving visceral disseminated VZV infection that emerged during remission induction therapy involving high-dose prednisolone (PSL), mycophenolate mofetil (MMF), and hydroxychloroquine (HCQ) for lupus nephritis. Additionally, we provide an overview of pertinent literature encompassing similar cases and report a literature review.

## Case report

**Fig. 1:**
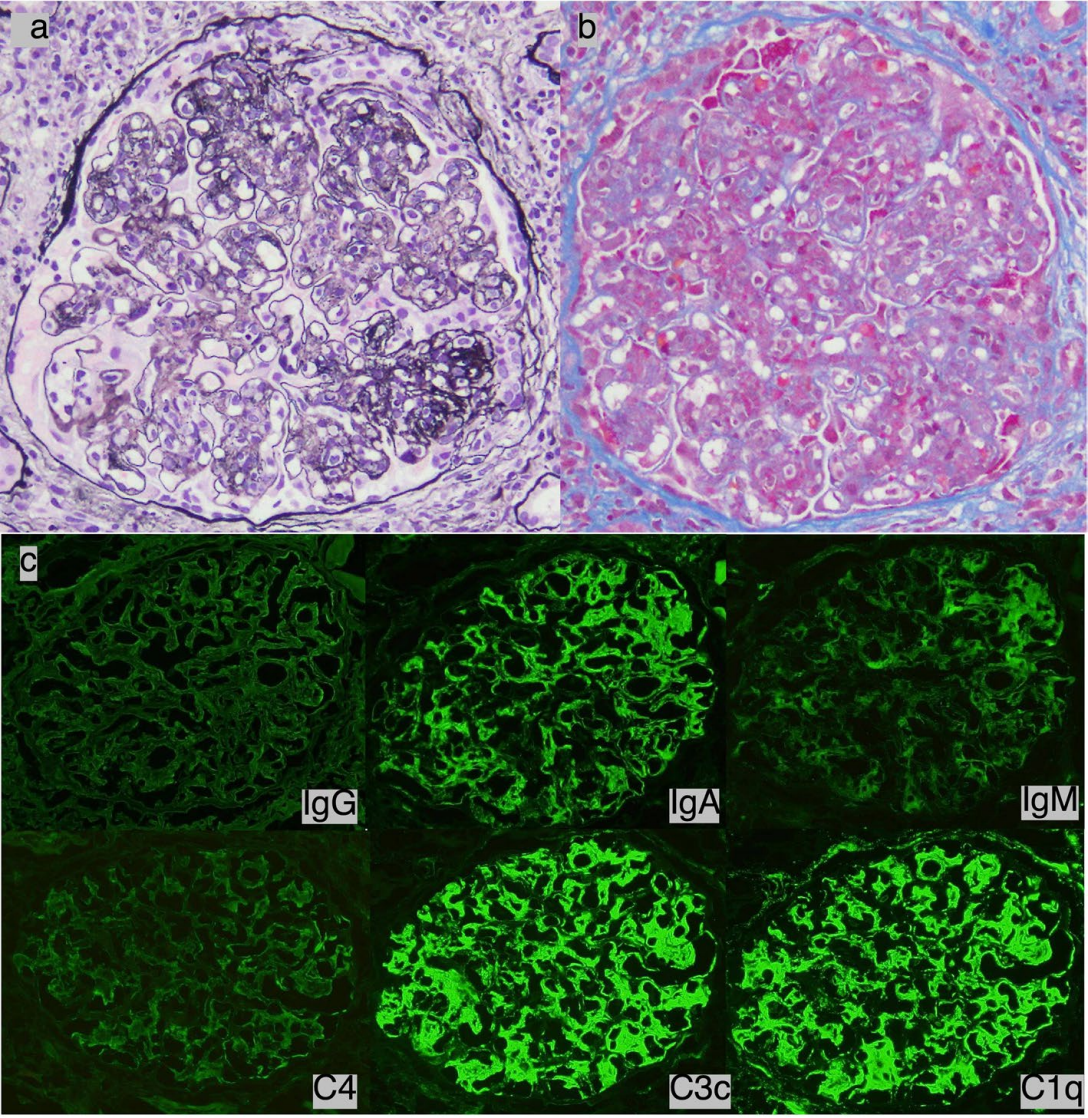
Light and immunofluorescent microscope findings of glomeruli in initial kidney biopsy before treatment shows mesangio-capillary proliferation with cellular crescent, and massive extensive deposits in mesangial and subendothelial regions. **a** PAM staining (×400). **b** Masson trichrome staining (×400). **c** Immunofluorescence (IgG, IgM, IgA, C1q, C3c, and C4) (original figures supplied by PCL Japan, Tokyo)

A 36-year-old Japanese man was referred to our hospital due to renal dysfunction. Upon admission, he exhibited multiple lymphadenopathy, epigastric pain, dry mouth, and skin rashs on his lower jaw that resembled acne vulgaris. The laboratory evaluations revealed anemia, positive results for anti-nuclear antibody, anti-double-stranded DNA antibody, and anti-Sm antibody, hypocomplementemia, renal dysfunction, proteinuria, and hematuria (Table 1). Cardiac ultrasonography identified pericardial effusion. Based on the diagnostic criteria outlined by the American College of Rheumatology (ACR) [15] and the Systemic Lupus International Collaborating Clinics (SLICC) [16], he was diagnosed with systemic lupus erythematosus (SLE). Kidney biopsy exhibited mesangio-capillary proliferation with cellular crescent and massive extensive deposits in mesangial and subendothelial regions. Although IgG- and C4-staining were weakly positive, fluorescence findings revealed a full-house pattern (Fig. 1), leading to a classification of lupus nephritis (LN) class IV as per the International Society of Nephrology/Renal Pathology Society (ISN/RPS) criteria [17].

**Table 1:** Laboratory findings before initial induction therapy

Hematology			Serology			Urinalysis		
WBC	7000	/μL	CRP	1.26	mg/dl	Protein	(4 +)	
RBC	4.19	× 10*6 /μL	RF	8.4	U/ml	Occult blood	(3 +)	
Hb	10.9	mg/dl	ASO	24	U/ml	Red blood cell	> 100	/HPF
Plt	211	× 10*3 /μL	IgG	3663	mg/dl	Protein content	3.06	g/g∙Cre
Seg	53	%	IgG4	225	mg/dl	NAG	27.4	IU/L
Lym	29	%	IgA	372	mg/dl	β2MG	189	μg/L
			IgM	73	mg/dl			
Biochemistry			C3	27	mg/dl			
TP	8.2	g/dl	C4	2	mg/dl			
Alb	2.7	g/dl	CH50	< 12.0	CH50 U/ml			
BUN	37.3	mg/dl	IL-2R	2760	U/ml			
Cre	1.9	mg/dl	IL-6	7.9	U/ml			
eGFR	34.4	ml/min/1.73m2	Antinuclear antibody	1280				
Na	135.7	mmol/L	Anti-ds-DNA-Ab	310	IU/ml			
K	4.8	mmol/L	Anti-PR3-ANCA (U/mL)	(−)				
Cl	103	mmol/L	MPO-ANCA (U/mL)	(−)				
Ca	9.9	mg/dl	Anti-GBM-Ab	(−)				
IP	3.7	mg/dl	Anti-SS-A-Ab	> 256				
AST	24	U/L	Anti-Sm-Ab	> 10				
ALT	19	U/L	Anti-cardiolipinβ2GP1- Ab	2.2	U/ml			
LDH	273	U/L	Lupus AC	37	Sec			
ALP	52	U/L	Coombs direct test	(+)				
Γ-GTP	44	U/L						
CK	48	U/L	Coagulation					
TB	0.3	mg/dl	PT-INR	1				
Ferritin	341	ng/ml	APTT	28.9	Sec			
			FDP	27.7	μg/dl

For the initiation of remission induction therapy, intravenous (IV) methylprednisolone was administered at a dosage of 1000 mg/day for three days, followed by oral prednisone at 60 mg/day (calculated as 1 mg/kg based on ideal body weight) for a duration of 4 weeks. Subsequent to the commencement of treatment, the epigastric pain resolved promptly and there was a gradual reduction in urinary protein excretion.

Three weeks post-treatment initiation, the patient commenced taking HCQ at a dosage of 200 mg and 400 mg on alternate days. During this period, CRP levels normalized and anti-double-stranded DNA antibody titers decreased to approximately one-quarter of pre-treatment values, indicating a reduction in disease activity. Concurrently, MMF was introduced at 500 mg while PSL dosage was tapered to 50 mg, subsequently escalated to 1000 mg after one week. As the dose escalated, the patient reported upper abdominal discomfort. While MMF-related side effects were suspected, the symptoms were not severe and the lymphocyte count remained unchanged from pre-treatment levels, with no reduction in lymphocytes indicative of decreased cell-mediated immunity, which supported the decision to continue treatment. Shortly thereafter, a painless ulcer manifested on the patient’s soft palate, raising concerns of disease resurgence.

After a few more days, on the 53rd day of hospitalization, the patient experienced a rapid onset of severe upper abdominal pain and difficulty swallowing was also present. At the same time or shortly thereafter, he reappearance of acneiform rash on the face. There was no lower back pain.

**Fig. 2:**
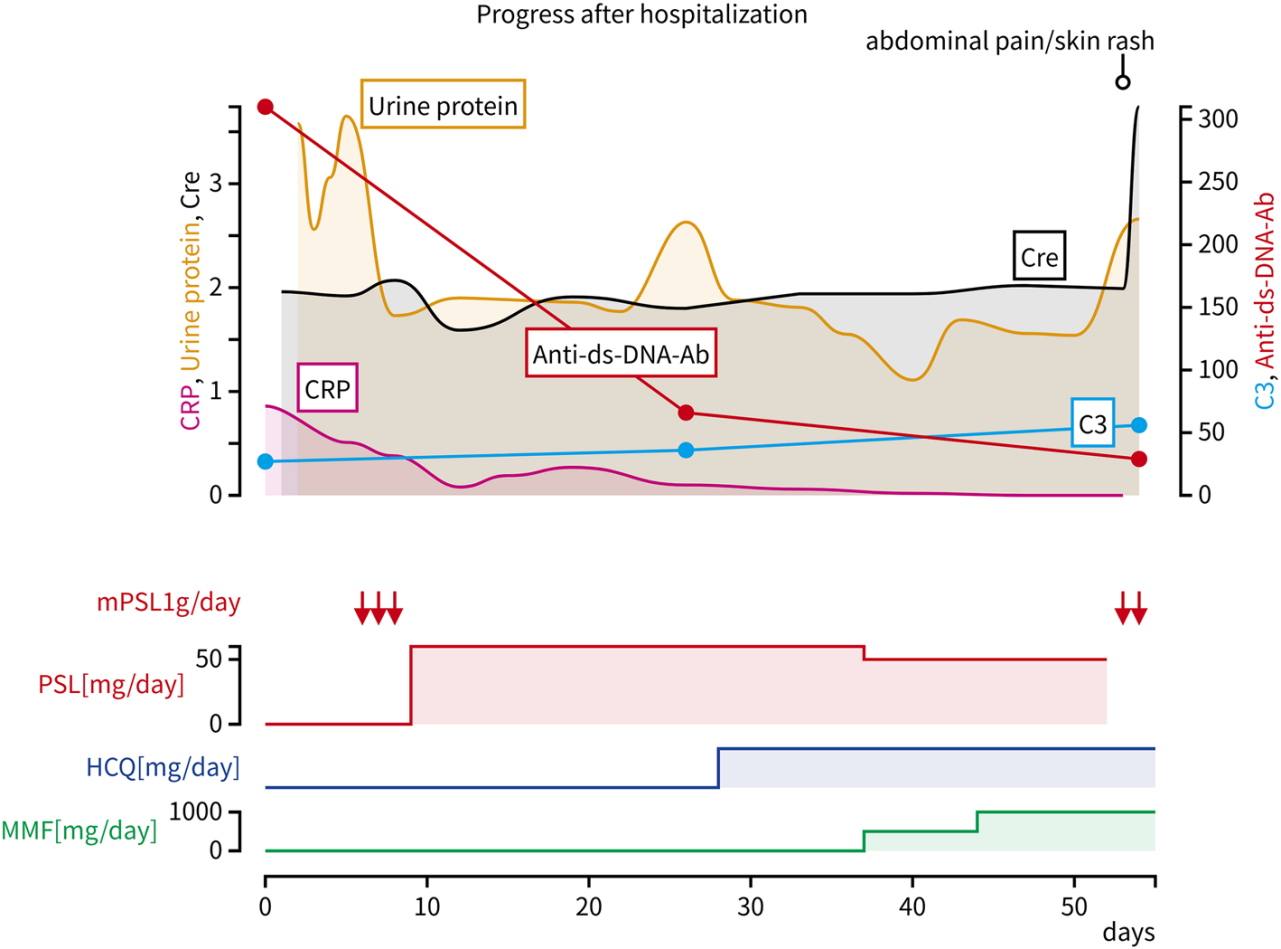
Timeline of the clinical course and duration of treatment

Laboratory examinations indicated deteriorating renal and hepatic function, alongside diminished platelet count. Suspecting inadequate disease control in SLE, additional glucocorticoid pulse therapy was started. However, the following day, clinical manifestations, and laboratory abnormalities rapidly deteriorated. Hyperferritinemia and hypertriglyceridemia, indicative of hemophagocytic lymphohistiocytosis (HLH), were observed alongside increasing liver and renal impairment and the new onset of disseminated intravascular coagulation (DIC), suggesting multiple organ failure (MOF) (Table 2). The patient developed widespread blistering predominantly on the trunk and face, coupled with oozing bleeding due to an aggravated bleeding tendency. While bone marrow biopsy was contemplated for HLH diagnosis, it had to be deferred due to severe coagulopathy. Considering a suspected infectious trigger for HLH, broad-spectrum antibiotics were initiated; however, the patient’s condition failed to improve, ultimately several hours later a fatal arrhythmia led to cardiac arrest during the course of MOF, on the 54th day of hospitalization (Fig. 2).

**Table 2:** Laboratory findings the next day after abdominal pain appeared

Hematology			Serology		
WBC	29,300	/μL	CRP	0.09	mg/dl
Hb	12.6	mg/dl	IgG	1200	mg/dl
Plt	52	× 10*3 /μL	C3	56	mg/dl
Seg	53	%	C4	2	mg/dl
Lym	23	%	CH50	< 12.0	CH50 U/ml
Myelo	8	%	IL-2R	623	U/ml
			Anti-ds-DNA-Ab	29	IU/ml
Biochemistry			Anti-cardiolipinβ2GP1-Ab	<1.2	U/ml
TP	5.8	g/dl	Lupus AC	240	Sec
Alb	3.2	g/dl	βD glucan	17.9	pg/ml
BUN	58	mg/dl	EBV-VCA-IgG	( +)	
Cre	3.78	mg/dl	EBV-VCA-IgM	(−)	
eGFR	16.2	ml/min/1.73m2	EBVA-Ab	( +)	
Na	135	mmol/L	CMVpp65antigen	(−)	
K	5.5	mmol/L			
Cl	103	mmol/L	Coagulation		
Ca	9.6	mg/dl	PT-INR	Unmeasurable	
IP	8.4	mg/dl	APTT	Unmeasurable	
AST	24	U/L	FDP	Unmeasurable	
ALT	945	U/L	Fibrinogen	Unmeasurable	
LDH	6838	U/L	D-dimer	> 800	μg/ml
AMY	732	U/L			
Γ-GTP	295	U/L	Urinalysis		
CK	114	U/L	Protein	(3+)	
TB	0.34	mg/dl	Occult blood	(3+)	
Ferritin	90,733	ng/ml	Red blood cell	> 100	/HPF
			Protein content	2.66	g/day

At autopsy, widespread skin petechiae were evident, while no significant bleeding lesions were identified. The effectiveness of SLE treatment was demonstrated by decreased systemic lymphadenopathy, reduced renal inflammatory cell infiltration, and absence of serositis. Chronic sialoadenitis with benign lymphoepithelial lesions was observed in the submandibular gland, indicating an overlap with Sjögren’s syndrome.

**Fig. 3:**
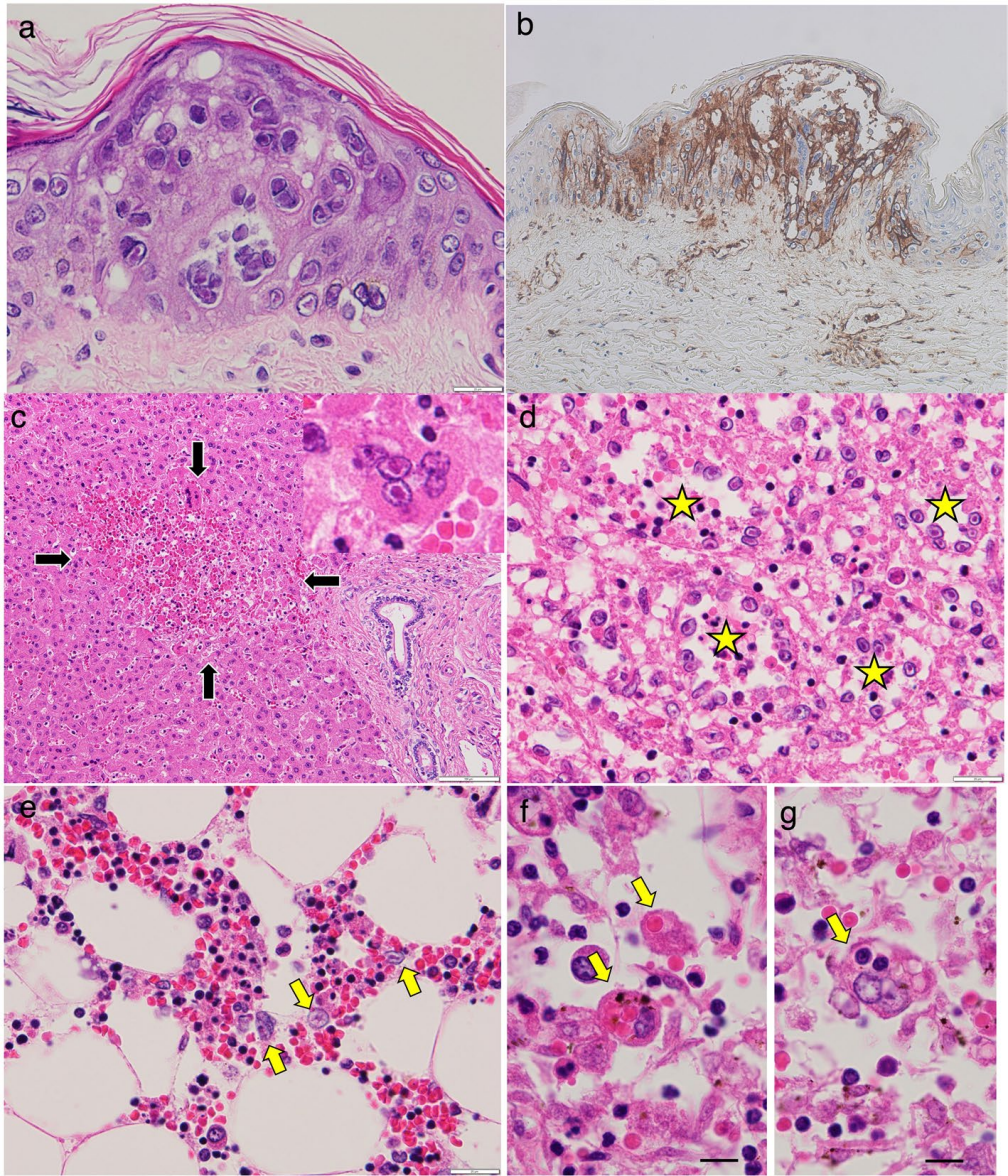
Microscopic findings at autopsy. Skin tissue shows multifocal clusters of cells with swollen nuclei and eosinophilic intranuclear inclusions (**a**). Such infected cells are clearly positive for varicella-zoster virus (VZV) (glycoprotein 1) along the cell membrane by immunohistochemistry (**b**). Also note positivity for VZV in blood vessels in the dermis (**b**). Liver tissue displays multifocal non-zonal necrosis of hepatocytes (**c**, arrows), with hepatocytes containing similar intranuclear inclusions in the peripheral areas of necrosis (inset of **c**). Similar intranuclear inclusions are confirmed in endothelial cells of sinuses (asterisks) of the spleen (**d**) and some of hematopoietic cells in the bone marrow (**e**, arrows). No areas with VZV infection display lymphocytic responses. Clear hemophagocytosis is confirmed in the subcapsular sinuses of lymph nodes; i.e., erythrophagocytosis in **f** (arrows) and lymphophagocytosis in **g** (arrow). Scale bars, 20 μm (**a**, **d**, **e**), 100 μm (**c**), and 10 μm (**f**, **g**)

Squamous epithelial cells in the skin, tongue, and esophagus displayed multifocal clusters of cells with swollen nuclei and large eosinophilic inclusions, some of which were associated with blisters. These lesions were thought to contribute to the patient’s dysphagia. By immunohistochemistry, affected cells in these lesions were positive for VZV antigen (glycoprotein-1) (clone C90.2.8) along the cell membranes (Fig. 3a). Herpes simplex virus (HSV)-1, HSV-2, and cytomegalovirus (CMV) were negative. The same-type intranuclear inclusions were detected in lymph nodes, spleen, bone marrow, liver, and salivary glands (Fig. 3b). In the liver, multiple foci of localized necrosis were observed around infected cells, which explained the clinical liver dysfunction (Fig. 3c). Therefore, the patient was diagnosed with visceral disseminated VZV infection.

In lymph nodes, hemophagocytosis was observed in macrophages in the subcapsular sinuses (Fig. 3d). Alongside the presence of fever, mild swelling of the spleen (167 g), hypertriglyceridemia, extremely severe hyperferritinemia, and absence of malignancy, the patient was diagnosed with secondary HLH associated with VZV infection [18]. Although VZV-related antibodies could not be retrieved, familial interview later confirmed that this was not his first infection with VZV.

## Discussion

Typically, VZV is a highly infectious herpesvirus that causes varicella during primary infection and herpes zoster during reactivation. In immunocompromised patients, recurrent VZV infection is not restricted to herpes zoster; it can also manifest as varicella. Visceral disseminated VZV infection, arising from either primary infection or reactivation, constitutes a rare but severe complication with a high mortality rate in immunosuppressed individuals [1–4]. Cell-mediated immunity is crucial for the prevention of VZV reactivation in adults [19]. Besides patients with blood disorders and organ transplants, individuals with autoimmune diseases such as SLE face a significantly heightened risk of VZV reactivation. Merayo-Chalico et al. identified lymphopenia (< 1000/μL) as an independent risk factor for severe infections in SLE patients, regardless of disease activity [20]. Treatment with glucocorticoids and immunosuppressants can further elevate the frequency and severity of VZV infections [2–7, 19–24].

MMF is a cornerstone in the management of lupus nephritis; however, several studies suggest that MMF usage may be a risk factor for VZV infection in SLE patients [9–11, 22]. Takeuchi T et al. reported on 418 Japanese patients diagnosed with lupus nephritis who used MMF. During treatment, it was reported that 10 of the 418 patients (3.34%) experienced herpes zoster, and one (0.24%) died from herpes zoster disseminated [26]. This risk has also been documented in transplant recipients and patients with blood disorders [4, 6, 27]. While visceral disseminated VZV infection is frequently reported in patients with blood disorders or organ transplants, recent years have seen an increase in such reports among SLE patients.

Including the present case, there have been 7 reported cases of visceral disseminated VZV infection in SLE patients from 2011 to 2023 [9–14] (Table 3). In all cases, abdominal pain and liver dysfunction preceded or coincided with the skin eruption. Concerning the time of onset, although some patients are on maintenance therapy, the onset typically occurs within a few weeks to several months after the initiation of remission induction therapy. There has been a reported case of fatalities even with early treatment, where elevated LDH levels were observed during the course of the disease. It is possible that secondary HLH developed and contributed to the fatal outcome similar to our case. This report is the first to show evidence of HLH occurring as a result of visceral disseminated VZV infection. Due to HLH complicated by visceral disseminated VZV infection, the present case’s general condition rapidly deteriorated.

**Table 3:** Summary of cases including our case

Number	Age/gender	Clinical manifestations	Medicine	Period from start of treatment for SLE to onset of symptoms	Treatment	Period from onset of symptoms to initiation of antiviral drugs	Outcome	Period from onset to death	Method of diagnosis
1	49/F	Abdominal pain liver dysfunction DIC MOF	MMF1.5 g/day PSL50mg/day	2 months	None	None	Death	Unknown (several days)	Autopsy
2	37/F	Abdominal pain liver dysfunction DIC systemic skin blisters(3 days later)	MMF1.5 g/day PSL40mg/day	2 months	Acyclovir intravenous immunoglobulin antibiotics taper PSL discontinued MMF	3 days	Survival		Systemic skin blisters Elevated copies of VZV DNA in blood
3	46/F	Abdominal pain liver dysfunction DIC systemic skin blisters(2 days later)	MMF1.5 g/day PSL40mg/day	2 months	Glucocorticoid pulse therapy continuous heparin infusion plasma exchange acyclovir	2 days	Death	7 days	Systemic skin blisters
4	23/M	Abdominal pain Herpes Zoster meningoencephalomyelitis	TAC 2 mg/day PSL40mg/day HCQ 400 mg/day	1 month	Acyclovir ganciclovir intravenous Immunoglobulin antibiotics antifungal drug mPSL plasma exchange cyclophosphamide	10 days	Survival		Herpes Zoster Elevated copies of VZV DNA in blood, cerebrospinal fluid, BALF
5	18/F	Skin blisters(at the same time as the following symptoms) lower back pain liver dysfunction DIC	TAC 2 mg/day PSL22.5 mg/day	3 years 9 months	acyclovir vidarabine	0 day	Survival		Skin blisters(Tzanck test)
6	48/F	Abdominal pain liver dysfunction lymphopenia thrombocytopenia systemic skin blisters(10 days later)	CyA 150 mg/day PSL5mg/day MTX12mg/week	13 years	Acyclovir	10 days	Survival		Systemic skin blisters Elevated copies of VZV DNA in blood(PCR)
our case	36/M	Abdominal pain liver dysfunction DIC MOF systemic skin blisters(1 days later)	MMF1.5 g/day PSL50mg/day HCQ200,500 mg/every other day	about 2 months	None (glucocorticoid pulse therapy, antibiotics)	None	Death	2 days	AUTOPSY

A review of prior case reports of visceral disseminated VZV infection in transplant and blood disorder patients reveals gastrointestinal symptoms, including intestinal obstruction, vomiting, and diarrhea, with abdominal pain being particularly common in most cases. These symptoms typically precede the skin lesions by several days (range 4–10 days, average 4.6 days) [3–7]. Until the rash emerges, visceral disseminated VZV is often misdiagnosed as abdominal pain of unknown etiology. The management of abdominal pain can be challenging and often necessitates the use of opioid analgesics. Severe abdominal pain may result from proliferation or inflammation caused by VZV in the celiac and mesenteric ganglia, although the precise mechanism remains unclear. In certain case series, numerous patients exhibited moderately or profoundly elevated aminotransferase levels and the majority had elevated pancreatic enzyme levels. The mortality rate was notably high, ranging from 20 to 50% [3–5]. Doki et al. [5] observed that most survivors commenced acyclovir treatment before or on the day of the skin lesion appearance, whereas non-survivors began treatment only after the lesions had appeared. Therefore, managing this serious complication necessitates early diagnosis and prompt or empirical medical intervention. Diagnostic methods included the identification of rashes, anti-VZV IgM/IgG antibodies, serum VZV-DNA, serum VZV antigens, PCR from lesion sites, and the Tzanck test [28].

As shown in Table 3, in addition to skin rash, an increase in VZV DNA copy number in the blood is associated with diagnosis. It has been reported that visceral disseminated VZV infection has an order of magnitude higher VZV DNA load in the blood compared to herpes zoster. Therefore, submitting a VZV DNA blood test is recommended, particularly for patients presenting with unexplained abdominal pain, elevated aminotransferase levels, and pancreatic enzymes approximately two months after commencing induction therapy. We believe this approach would facilitate early diagnosis and treatment, preceding the onset of skin rash.

Although no definitive treatment has been established, initiating treatment with adequate doses of acyclovir as early as possible and reducing the dose of immunosuppressive therapy are considered crucial. In certain cases, intravenous high-dose immunoglobulin therapy (IVIg) was administered as part of the treatment regimen for patients with severe infections and hypogammaglobulinemia [10, 28].

Compared to other cases, the case we encountered had a notably rapid disease progression, with the interval from the onset of severe upper abdominal pain to death being approximately two days. However, when the patient reported mild upper abdominal discomfort upon increasing the MMF dose, measuring VZV DNA in the blood might have allowed for some form of intervention.

## Conclusion

Here we have reported a case study detailing the fatal outcome of visceral disseminated VZV infection with secondary HLH in a Japanese patient with lupus nephritis. It is crucial to emphasize the awareness of this grave complication, particularly in cases patients are undergoing concurrent high-dose glucocorticoid therapy alongside MMF for SLE.

The progression of this lethal condition typically commences with abdominal pain, trailed by the emergence of a skin rash after several days. However, the administration of antiviral medication post-rash manifestation may be too late. Therefore, for early diagnosis and treatment, it is necessary to measure VZV-DNA in case exhibiting potential susceptibility to this disease.
